# Safety, Pharmacokinetics, and Pharmacodynamics of Single-Dose Programmed Cell Death Protein 1 Inhibitor, Budigalimab, in People With HIV-1 With Antiretroviral Therapy–Suppressed Viral Load

**DOI:** 10.1093/infdis/jiag142

**Published:** 2026-04-07

**Authors:** Tanaya R Vaidya, Till Geib, Jacob P Lalezari, Christopher Bettacchi, Cynthia C Brinson, Peter J Ruane, Heide Betman, Fei Zhou, Preethi Krishnan, Nael M Mostafa

**Affiliations:** AbbVie Inc., North Chicago, Illinois, USA; AbbVie Inc., North Chicago, Illinois, USA; Quest Clinical Research, SanFrancisco, California, USA; North Texas Infectious Disease Consultants, Dallas, Texas, USA; Central Texas Clinical Research, Austin, Texas, USA; Ruane Clinical Research, Los Angeles, California, USA; AbbVie Inc., North Chicago, Illinois, USA; AbbVie Inc., North Chicago, Illinois, USA; AbbVie Inc., North Chicago, Illinois, USA; AbbVie Inc., North Chicago, Illinois, USA

**Keywords:** budigalimab, anti–PD-1 monoclonal antibody, HIV

## Abstract

**Background:**

Blockade of inhibitory immune checkpoint receptor programmed cell death protein 1 (PD-1) on target immune cells is associated with improved HIV-specific immune function and activation of latent HIV. This randomized, placebo-controlled, Phase 1b study assessed low doses of investigational anti–PD-1 monoclonal antibody, budigalimab, for safety, tolerability, pharmacokinetics, and pharmacodynamics in people with HIV (PWH) on antiretroviral therapy.

**Methods:**

Participants received single doses of budigalimab 10 mg subcutaneous (SC), 20 mg SC, 10 mg intravenous (IV), or placebo (n = 8 per arm) and were followed for 24 weeks.

**Results:**

Of 32 randomized participants, 22 reported adverse event(s) (AE); most (n = 19) were grade ≤2 and no grade ≥4 AE or treatment-related serious AE. Two participants reported a non-treatment-related grade 3 AE (placebo, n = 1 pneumonia; 10 mg IV, n = 1 elevated aspartate aminotransferase). One reversible immune-related AE (grade 2 lichenoid keratosis) was reported (20 mg SC). Geometric mean maximum serum concentrations were 0.37, 1.57, and 3.2 µg/mL with 10 mg SC, 20 mg SC, and 10 mg IV, respectively. Drug exposure with 20 versus 10 mg SC dosing was more than dose proportional and less variable. Subcutaneous bioavailability was approximately 53%–62%. The PD-1 receptor saturation was ≥95% in most participants (median duration: 20 mg SC, 42 days; 10 mg SC, 14 days; 10 mg IV, 35 days).

**Conclusions:**

Findings suggest an acceptable safety profile for single-dose budigalimab in PWH, with a favorable pharmacokinetic profile for 20 mg SC and 10 mg IV. Further evaluation as a potential component of an HIV treatment is underway.

## Teaser

Budigalimab, an investigational anti–programmed cell death protein 1 monoclonal antibody, is a potential treatment for HIV. Assessment of a single low dose (≤20 mg) of budigalimab in people with HIV demonstrated an acceptable safety profile and favorable pharmacokinetics and pharmacodynamics with subcutaneous or intravenous injection.

Programmed cell death protein 1 (PD-1), a cell surface protein predominantly expressed on activated T cells, is an inhibitory immune checkpoint receptor that has been identified as having a role in immune exhaustion that contributes to the persistence of some chronic viral diseases, including HIV and hepatitis C virus (HCV) [1–6]. In HIV infection, preclinical and clinical data suggest that antibody blockade of PD-1/PD-1 ligand binding may play a role in promoting control of chronic infection and viral clearance through multiple mechanisms. Interruption of PD-1 interaction has been associated with survival, proliferation, and functional restoration of exhausted HIV-specific CD8^+^ T cells [4, 7] and induction or enhancement of latent HIV activation in infected CD4^+^ T cells [8–10].

Budigalimab is a humanized, recombinant immunoglobulin gamma 1 L234A L235A monoclonal antibody (mAb) that binds to cell surface–expressed PD-1 and blocks ligand-receptor interaction [11]. Budigalimab has been investigated in early-phase trials in oncology patients based on the role of PD-1 inhibitors in promoting T-cell–mediated immune responses to solid tumors [11–13]. Given the evidence supporting anti–PD-1 mAbs in viral control, budigalimab is a potential treatment for HIV. The doses under study in HIV treatment are substantially lower than those used in oncology and are derived from modeling results from budigalimab and other anti–PD-1 mAb studies [14]. The use of lower-dose anti–PD-1 mAbs in chronic infection is supported by studies in hepatitis B virus and HCV [6, 15].

The current study (ClinicalTrials.gov: NCT04799353) is a phase 1b randomized, double-blind study to investigate low-dose (≤20 mg) budigalimab and evaluate subcutaneous (SC) and intravenous (IV) routes of administration. The main study objectives were to evaluate the safety, tolerability, pharmacokinetics (PK), immunogenicity, and pharmacodynamics after SC and IV administration of a single dose of budigalimab in people with HIV (PWH) on suppressive antiretroviral therapy (ART). Exploratory biomarkers were examined to assess biomarker signatures of potential immunomodulatory effects of budigalimab.

## METHODS

This report was prepared following CONSORT reporting guidelines [16].

### Study Design and Treatments

Study M19-972 was a phase 1b, randomized, double-blind, and placebo-controlled study of budigalimab evaluating safety, PK, and pharmacodynamics (PD) following a single IV or SC dose in PWH with ART-suppressed viral load. The study enrolled participants at 7 study sites in the United States, including Puerto Rico. Eligible participants were randomly assigned in parallel (1:1:1:1) to receive placebo (IV and SC), budigalimab 10 mg SC, budigalimab 20 mg SC, or budigalimab 10 mg IV. A placebo was also administered in active study treatment arms for the alternate route of administration to maintain double blinding. Participants received a single dose of active study treatment or placebo on study day 1 and remained on their ART regimen during the 24-week follow-up. Low doses were selected with an expectation of a more favorable safety margin in comparison with oncologic doses, specifically targeting the doses to be lower than 1 mg/kg. Moreover, a 10 mg IV dose was being evaluated in a multiple-dose study in PWH undergoing ATI in a parallel phase 1b study (M19-939, ClinicalTrials.gov: NCT04223804) [17], thus leading to the selection of this dose level for evaluation in the present study. A 10 mg SC dose was selected to determine the SC bioavailability of budigalimab. An additional dose of 20 mg SC was selected to approximately match projected exposures with the 10 mg IV dose and to determine the dose proportionality with the 10 mg SC dose.

The primary endpoints were study drug–related grade 3 or higher adverse events (AEs), study drug–related immune-related AEs (irAEs), and PK parameters. Immunogenicity and pharmacodynamic endpoints were also assessed. There were no analytical treatment interruptions, and participants were required to be on ART for the duration of the study.

All sites used a central institutional review board (Advarra, Inc., Columbia, MD) to approve the study protocol, informed consent, and participant information. The study was conducted in accordance with International Council for Harmonisation guidelines and applicable regulations, guidelines, and principles having their origin in the Declaration of Helsinki. Written informed consent was obtained for each participant before screening or study-specific procedures.

### Participants

Eligible participants were male or female, 18–65 years old, with a positive anti-HIV antibody test and on ART for at least 12 months prior to screening. Plasma HIV-1 RNA levels were below the lower limit of quantitation (20 copies/mL) at screening and for at least 6 months prior to screening, and CD4^+^ cell counts were ≥450 cells/µL at screening and at least once during the 12 months prior to screening. Exclusion criteria included active or history of AIDS-defining illness; active hepatitis B virus or HCV infection; active, suspected, or history of malignancy; active or history of autoimmune disease requiring systemic treatment; a current ART regimen containing maraviroc; and previous therapy/exposure to any immune checkpoint inhibitor.

### Safety Assessments

Safety was assessed throughout the study by vital signs, physical examination, clinical laboratory tests (including hematology and metabolic panels, thyroid function, hemolysis, plasma HIV-1 RNA, and CD4^+^ cell counts), and monitoring of AEs, which were coded using the Medical Dictionary for Regulatory Activities (MedDRA, version 25.0). Investigators assessed whether an AE had a reasonable possibility of a relationship to the study drug. Adverse events of special interest (AESIs) were irAEs, injection-site reactions, infusion-related AEs, and hepatotoxicity. Virologic failure was defined as confirmed (2 consecutive) plasma HIV-1 RNA ≥200 copies/mL.

### Pharmacokinetic, Immunogenicity, and Pharmacodynamic Assessments

Serial blood samples for measurement of serum budigalimab concentration were collected prior to dosing and ≤15 minutes, 2 hours (±15 minutes), and 4 hours (±15 minutes) after dosing on study day 1. Samples were also collected on days 2 and 3, followed by weeks 1, 2, 4, 6, 8, 12, and 24. Serum concentrations of budigalimab were measured with a validated electrochemiluminescence immunoassay. The validation parameters of the analytical method were as follows: the lower limit of quantitation was 27.8 ng/mL in 100% human serum. The dynamic range of the assay was 27.8 to 4440 ng/mL, with a 5-parameter logistic model (Marquardt) and 1/*Y*^2^ weighting used to fit the data. The intra-run precision (% CV) and accuracy (% Bias) were ≤19.4 and between −9.1 and 14.1, respectively. The inter-run precision (% CV) and accuracy (% Bias) were ≤10.5 and between 0.1 and 2.9, respectively. The dilution linearity of the assay was demonstrated up to 2.0 mg/mL. Pharmacokinetic parameters of budigalimab were calculated using noncompartmental methods with the software Phoenix® WinNonlin® version 8.0 (Certara L.P. [Pharsight], St Louis, MO) and included maximum observed serum concentration (*C*_max_), time to *C*_max_ (*T*_max_), area under the serum concentration–time curve (AUC), and terminal phase elimination half-life (*t*_1/2_). The study also calculated budigalimab bioavailability following SC administration compared with IV administration. The immunogenicity of budigalimab was assessed by detecting antidrug antibodies (ADAs) using a validated titer-based electrochemiluminescence immunoassay.

Pharmacodynamic analysis included PD-1 receptor saturation assessment, with samples taken on days 1 (before dosing) and 2 and weeks 1, 2, 4, 6, 8, 12, and 24. Receptor saturation levels were assessed using flow cytometry, as previously described [18]. A custom validated assay performed at a single center (LabCorp, Indianapolis, IN) examined PD-1 receptor saturation on memory T-cell subtypes using a panel of CD28-FITC, CD279-PE, CD3-PERCP, CD95-BV421, CD8-BV510 (all from Becton, Dickinson and Co., Franklin Lakes, NJ), CD4-BV605 (BioLegend, San Diego, CA), and OX40-AF647 (AbbVie Inc., North Chicago, IL). To account for variability in gating with a flow cytometry–based assay, nearly complete PD-1 receptor saturation was defined as receptor saturation ≥95% on CD3^+^/8^+^ cells.

### Biomarker Analyses

A description of the methods for biomarker analysis can be found in the Supplementary Material.

### Statistical Methods

All participants receiving any amount of budigalimab or placebo were included in the analyses, and data were summarized by treatment arm and by sampling time for PK/PD parameters. The bioavailability of the SC versus IV doses and dose proportionality of 20 mg SC versus 10 mg SC doses were investigated using analysis of covariance. No power calculations for sample size considerations were performed for this descriptive study. A sample size of 8 participants per arm is within the range of those commonly used in phase 1 studies. Statistical analyses were performed using SAS (SAS Institute, Inc., Cary, NC).

## RESULTS

### Study Population and Participant Disposition

Of 67 participants screened, 35 failed screening, and 32 were randomized, 8 to each of 4 treatment groups. All participants completed the study treatment and the study. One participant in each of the 10 and 20 mg SC dose groups had all budigalimab concentration measures below the lower limit of quantitation and incomplete PD-1 receptor saturation; these participants were excluded from PK and PD analysis but included in the safety analysis. No deviation from protocol was found, and the observations remain unexplained.

All participants were male, most were White and non-Hispanic/Latino, and the mean age across all participants was 47 years (Table 1). At baseline, the mean duration of HIV diagnosis was 14.1 years (range, 2–40 years), and the mean duration of viral suppression on ART was 12.8 years (range, 2–32 years). The mean CD4^+^ T-cell count at baseline was 743.5 cells/µL (range, 404–1322 cells/µL), and a total of 2 participants reported a CD4^+^ T-cell nadir of <200 cells/µL.

**Table 1.** Baseline Demographics and Characteristics

	Placebo	Budigalimab		
	n = 8	10 mg SC n = 8	20 mg SC n = 8	10 mg IV n = 8
Sex—male, n (%)	8 (100)	8 (100)	8 (100)	8 (100)
Race, n (%)				
Asian	0	0	0	2 (25.0)
Black/African American	1 (12.5)	3 (37.5)	0	0
White	6 (75.0)	5 (62.5)	7 (87.5)	5 (62.5)
Other, include multiple	1 (12.5)	0	1 (12.5)	1 (12.5)
Ethnicity, n (%)				
Hispanic/Latino	3 (37.5)	5 (62.5)	3 (37.5)	1 (12.5)
Not Hispanic/Latino	5 (62.5)	3 (37.5)	5 (62.5)	7 (87.5)
Age (y), mean (SD)	48.8 (12.6)	46.4 (13.5)	46.6 (12.2)	45.5 (13.2)
BMI (kg/m^2^), mean (SD)	27.5 (3.0)	27.8 (3.3)	28.9 (4.1)	26.1 (4.6)
Duration of HIV-1 diagnosis (y), mean (SD)	15.1 (9.2)	13.3 (8.9)	13.6 (7.4)	14.4 (12.8)
Duration of viral suppression on ART (y), mean (SD)	15.4 (8.5)	11.5 (9.0)	12.6 (6.8)	11.8 (11.0)
ART regimen at study entry, n (%)				
PI based	0	0	0	1 (12.5)
INSTI based	7 (87.5)	4 (50)	5 (62.5)	7 (87.5)
Other	1 (12.5)	4 (50)	2 (25)	0
Missing	0	0	1 (12.5)	0
CD4^+^ T-cell nadir				
<200 cells/µL	1 (12.5)	0	0	1 (12.5)
<350 cells/µL	3 (37.5)	0	0	2 (25.0)
CD4^+^ T-cell count (cells/µL), mean (SD)	785.6 (243.0)	766.6 (325.5)	760.9 (148.2)	660.8 (104.1)
%PD-1 (CD3^+^/CD8^+^), mean (SD)	32.8			
	(14.7)	42.6 (11.1)	36.7 (11.2)	39.2 (18.4)

Abbreviations: ART, antiretroviral therapy; BMI, body mass index; INSTI, integrase strand transfer inhibitors; IV, intravenous; PD-1, programmed cell death protein 1; PI, protease inhibitors; SC, subcutaneous.

### Safety

Overall, 22 (69%) participants reported any AE during the study, including 5/8 (63%) participants receiving a placebo (Table 2). Most AEs had a maximum severity of grade 1 (n = 13) or grade 2 (n = 7), and there were no grade 4 AEs or deaths. Two (6%) participants reported a grade 3 AE, both deemed by the investigator as not related to the study drug (placebo, n = 1 pneumonia; 10 mg IV, n = 1 elevated aspartate aminotransferase [AST]). The most common AEs (≥5% of all participants) were COVID-19, pyrexia (n = 4 each), diarrhea, fatigue, headache (n = 3 each), and myalgia (n = 2). Treatment-related AEs were reported in 9/32 (28%) of participants, with no treatment-related AE being reported in >3 (9%) participants across all study arms. A single irAE was reported in 1 participant receiving budigalimab 20 mg SC; treatment-related nonserious grade 2 lichenoid keratosis developed on day 34 and resolved on day 52. One hepatic-related AESI of nonserious grade 3 AST elevation (mentioned above; days 14–21) was reported in association with a grade 1 nonserious elevation of alanine aminotransferase in a participant receiving the 10 mg IV dose. This event was deemed not to be related to the study drug due to an alternate etiology of excessive supplement use. There were no reactions related to the route of administration and no virologic failures.

**Table 2.** Safety Assessment

	Placebo	Budigalimab			Total
	n = 8 n (%)	10 mg SC n = 8 n (%)	20 mg SC n = 8 n (%)	10 mg IV n = 8 n (%)	N = 32 n (%)
Any AE	5 (62.5)	6 (75.0)	5 (62.5)	6 (75.0)	22 (68.8)
Grade 1	2 (25.0)	4 (50.0)	3 (37.5)	4 (50.0)	13 (40.6)
Grade 2	2 (25.0)	2 (25.0)	2 (25.0)	1 (25.0)	7 (21.9)
Grade 3	1 (12.5)	0	0	1 (25.0)	2 (6.3)
Serious AE	1 (12.5)	0	0	0	1 (3.1)
TR-AE^a^	2 (25.0)	1 (12.5)	2 (25.0)	4 (50.0)	9 (28.1)
Grade ≥3 TR-AE	0	0	0	0	0
Serious TR-AE	0	0	0	0	0
irAE	0	0	1 (12.5)^b^	0	1 (3.1)
Hepatic-related AESI	0	0	0	1 (12.5)^c^	1 (3.1)
Death	0	0	0	0	0
Most frequent AEs (≥5% of participants)					
COVID-19	1 (12.5)	1 (12.5)	2 (25.0)	0	4 (12.5)
Pyrexia	1 (12.5)	2 (25.0)	0	1 (12.5)	4 (12.5)
Diarrhea	0	1 (12.5)	0	2 (25.0)	3 (9.4)
Fatigue	0	2 (25.0)	0	1 (12.5)	3 (9.4)
Headache	1 (12.5)	1 (12.5)	0	1 (12.5)	3 (9.4)
Myalgia	0	2 (25.0)	0	0	2 (6.3)
Most frequent TR-AEs (≥5% of participants)					
Diarrhea	0	1 (12.5)	0	2 (25.0)	3 (9.4)
Fatigue	0	1 (12.5)	0	1 (12.5)	2 (6.3)

Abbreviations: AE, adverse event; AESI, adverse event of special interest; irAE, immune-related AE; IV, intravenous; SC, subcutaneous; TR-AE, treatment-related AE.

^a^As assessed by the investigator.

^b^Deemed to be treatment related.

^c^Not deemed to be treatment related owing to the alternative etiology of excessive supplement use.

### Pharmacokinetics, Immunogenicity, and Pharmacodynamics

**Figure 1.**
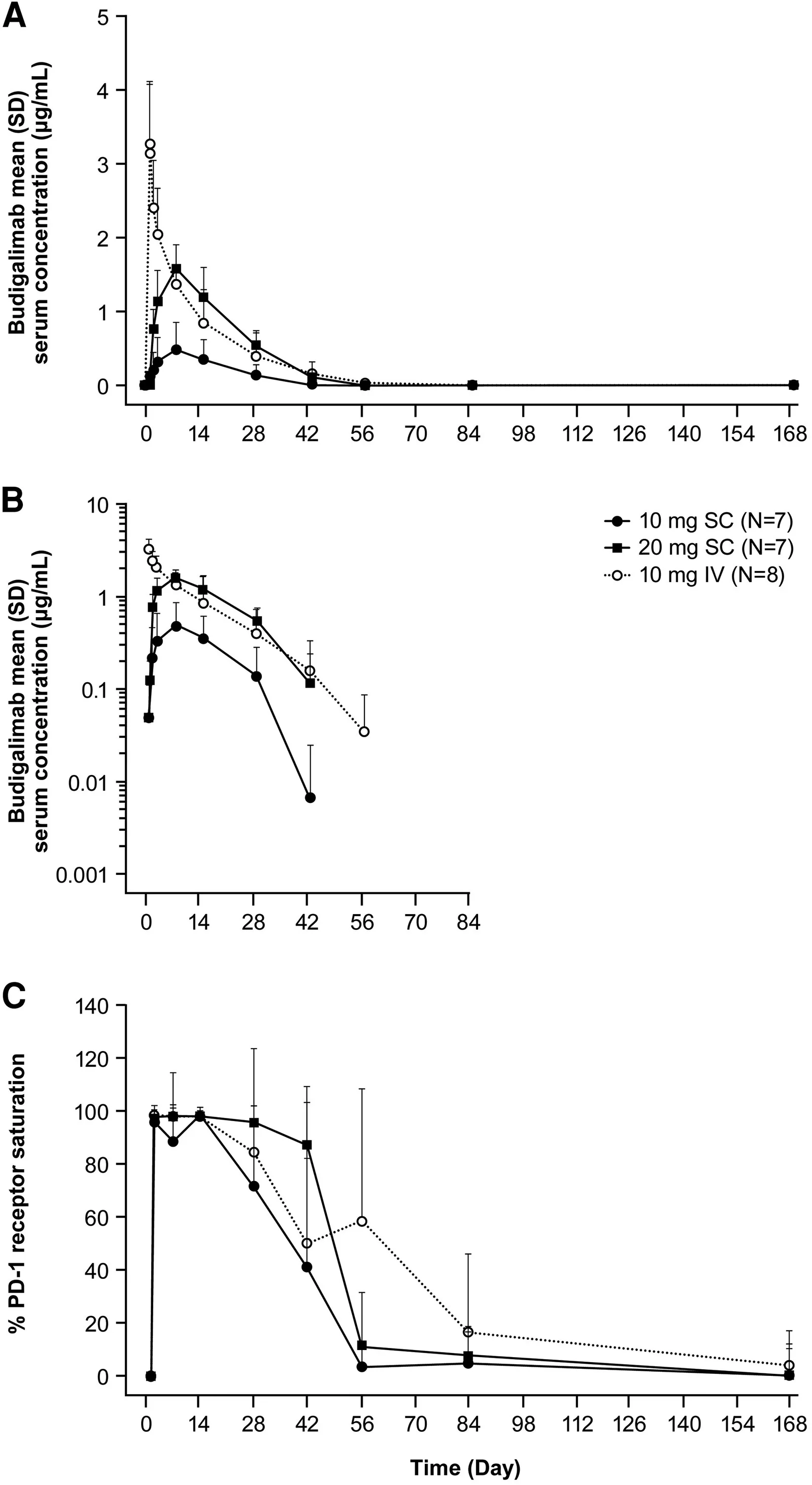
(*A*) Linear scale and (*B*) semi-logarithmic scale of mean (SD) serum budigalimab concentration–time profiles following SC and IV administration and (*C*) mean (SD) PD-1 receptor saturation following budigalimab administration. One participant in each of the 10 mg SC and 20 mg SC dose groups had all budigalimab concentrations below the lower limit of quantitation and incomplete PD-1 receptor saturation; these participants were excluded from the analysis. Abbreviations: IV, intravenous; PD-1, programmed cell death protein 1; SC, subcutaneous.

Mean budigalimab serum concentration–time profiles by dose group are presented in Figure 1*A* and 1*B*. Following administration of a single dose, the geometric mean *C*_max_ with budigalimab 20 mg SC administration was 1.57 µg/mL, while that with 10 mg IV administration was 3.2 µg/mL (Table 3). The geometric mean *C*_max_ with 10 mg SC administration (0.37 µg/mL) was approximately 10-fold lower than with 10 mg IV administration. Median *T*_max_ was approximately 8 days, 6 days, and 0.25 hours for 10 mg SC, 20 mg SC, and 10 mg IV dosing, respectively. The mean apparent terminal elimination half-life was 6.5, 9, and 8 days for 10 mg SC, 20 mg SC, and 10 mg IV, respectively. On the basis of *C*_max_ and AUC*_t_*, drug exposure with 20 mg SC dosing was more than dose-proportional relative to 10 mg SC dosing, and exposures were more variable with 10 mg SC dosing (coefficient of variation >70%) than with 20 mg SC dosing (Supplementary Table 1 and Supplementary Figure 1). Analysis of covariance for bioavailability assessment of SC administration demonstrated that following SC administration of budigalimab 10 mg, the bioavailability was approximately 53% (Table 4). Following SC administration of budigalimab 20 mg, the bioavailability was approximately 62%.

**Table 3.** Pharmacokinetic Parameters Following Single SC and IV Doses of Budigalimab

	Budigalimab		
PK Parameters (unit)	10 mg SC (n = 7)^a^	20 mg SC (n = 7)^a^	10 mg IV (n = 8)
*C*_max_^b^ (µg/mL)	0.37 (0.49, 74.0)	1.57 (1.61, 20.3)	3.20 (3.30, 26.0)
*T*_max_^c^ (h)	194.9 (146.8–11.6)	146.5 (46.3–336.0)	0.25 (0.25–4.0)
*t*_1/2_^d^ (d)	6.47 (0)^e^	9.32 (4.03)^f^	8.02 (3.79)
AUC*_t_*^b^ (µg d/mL)	5.69 (8.95, 93.9)	34.0 (34.8, 23.3)	30.4 (34.2, 49.4)
AUC_inf_^b^ (µg d/mL)	23.9 (23.9, –)^e^	43.1 (43.3, 12.7)^g^	32.4 (36.3, 48.8)

Abbreviations: AUC, area under the serum concentration–time curve; AUC_inf_, AUC from time 0 to infinite time; AUC*_t_*, AUC from time 0 to the time of the last measurable concentration; *C*_max_, maximum observed serum concentration; IV, intravenous; SC, subcutaneous; *T*_max_, time to reach *C*_max_; *t*_1/2_, terminal phase elimination half-life.

^a^One participant in the SC groups had all budigalimab concentrations below the lower limit of quantitation and was excluded from the analysis.

^b^Geometric mean (mean, % coefficient of variation).

^c^Median (minimum through maximum).

^d^Harmonic mean (pseudo-standard deviation).

^e^Calculable for n = 1 participant.

^f^Calculable for n = 4 participants.

^g^Calculable for n = 3 participants.

**Table 4.** Budigalimab Bioavailability SC Versus IV: Ratio of Central Values (90% CI) for AUC for Budigalimab Following Single SC Versus IV Dosing

Group	Test vs Reference	PK Parameter	Test	Reference	Estimate	90% Confidence Interval
Budigalimab 10 mg SC	10 mg SC vs 10 mg IV	AUC_inf_ (µg d/mL)	23.9	32.4	0.53	0.23–1.24
Budigalimab 20 mg SC	20 mg SC vs 10 mg IV	AUC_inf_/dose (µg d/mL/mg)	2.15	3.24	0.62	0.38–1.00

Abbreviations: AUC, area under the serum concentration–time curve; AUC_inf_, AUC from time 0 to infinite time; IV, intravenous; SC, subcutaneous.

Treatment-emergent ADAs were observed in 8/32 participants across all dose groups in the study. Of the 8 participants, 5 were from the 10 mg SC group and 3 were from the 10 mg IV group. The baseline prevalence of ADA was 0% across all groups. Budigalimab exposures of participants with positive ADA titers generally overlapped with or were slightly higher than those without ADA titers for the 10 mg IV dose group. However, the exposures of participants with positive ADA titers for the 10 mg SC dose trended lower than those without ADA titers (Supplementary Figure 2).

Baseline expression of PD-1 on CD4^+^ and CD8^+^ T cells and subsets was comparable across doses. Analysis of PD-1 receptor saturation on CD8^+^ T cells found ≥95% PD-1 receptor saturation lasted 14 days (n = 6) and 28 days (n = 1) in the 10 mg SC group; 28 days (n = 2) and 42 days (n = 5) in the 20 mg SC group; and 14 days (n = 1), 28 days (n = 3), and 42 days (n = 1) to 56 days (n = 3) in the 10 mg IV group. Mean percent PD-1 receptor saturation versus time plots stratified by the treatment arm is depicted in Figure 1*C*. PD-1 receptor saturation was comparable across CD4^+^ and CD8^+^ T cells and subsets. PD-1 receptor saturation was not observed in samples from placebo-treated participants.

### Biomarker Analyses

There was no apparent Ki67^+^ proliferative burst on CD8^+^ T-cell memory population (CD45RO^+^ cells) post budigalimab or placebo dosing (Supplementary Figure 3). No changes in HLA-DR, Granzyme B, or memory subset distribution were observed in this period (data not shown). Cell-associated unspliced RNA was evaluated at W1 and W2 after the first dose, and no significant change (Mann–Whitney test) in cell-associated unspliced RNA was observed (Supplementary Figure 4). In line with these observations, no changes in HIV- or Cytomegalovirus (CMV)-specific T-cell responses as measured by IFNγ ELISpot assay were apparent (Supplementary Figure 5).

## DISCUSSION

This Phase 1b study evaluated the safety and PK of single low doses of the PD-1 inhibitor budigalimab administered SC or IV and found all doses to have an acceptable safety profile in all treatment arms with a relatively low frequency and severity of AEs in PWH on suppressive ART. There were no study drug–related grade ≥3 AEs; 1 nonserious, reversible irAE of grade 2 lichenoid keratosis; and no virologic failures during the study.

Although current HIV treatments are safe and effective, several carry known chronic concerns. Many HIV regimens are available, and the safety risk for any new potential treatment must be acceptable within this context. A previous Phase 1b study of 2–10 mg doses of budigalimab (M19-939, NCT04223804) reported 3 grade 3 AEs and 1 serious AE, but none were deemed related to treatment [17]. The safety profile observed in this study of low dose (≤20 mg) budigalimab administered SC or IV was acceptable, and toxicity was manageable in adult PWH participants who were virologically suppressed on ART. The severity and types of reported AEs were as expected based on known modes of action and consistent with those occurring in this study population. No new safety signals were observed.

One case of a nonserious, reversible irAE of lichenoid keratosis occurred in a participant receiving 20 mg SC dosing and resolved with topical steroids and diphenhydramine within 19 days. The participant was a 54-year-old Asian male with well-controlled HIV (using darunavir/ritonavir plus emtricitabine/tenofovir disoproxil fumarate) and a medical history of environmental allergies, asthma, hypertension, degenerative joint disease, and gastroesophageal reflux disease. A family history of environmental dermatologic allergies to food was reported. Budigalimab exposures for this participant were not notably higher than those of other participants within the same dose group.

Pharmacokinetic analyses revealed that exposures associated with the 20 mg SC dose were less variable and more than dose-proportional relative to the 10 mg SC dose. Following SC administration of budigalimab 10 and 20 mg, bioavailability was approximately 53% and 62%, which is within the expected range of SC bioavailability for IgG1 monoclonal antibodies [20]. The terminal phase elimination half-life was 6.5 to 9 days across 10 and 20 mg SC and 10 mg IV. No ADAs were detected in the 20 mg SC dose group, while positive ADA titers were detected in 5/8 participants in the 10 mg SC group and 3/8 participants in the 10 mg IV group. Positive ADA titers were associated with lower exposures in the 10 mg SC dose group, while no apparent impact of ADA on budigalimab exposures was observed for the 10 mg IV dose group. Analysis of PD-1 receptor saturation on CD8^+^ T cells noted ≥95% PD-1 receptor saturation ranging from 14 to 56 days (median of 35 days) with 10 mg IV dosing. In the 20 and 10 mg SC groups, ≥95% receptor saturation was observed for a median duration of 42 days and 14 days, respectively. Overall, these initial findings suggest that budigalimab doses of 20 mg SC or 10 mg IV have favorable PK/pharmacodynamic profiles and potentially durable PD-1 receptor saturation.

A limitation of this study was the low number of participants for safety analysis; however, this number was adequate for PK/PD analyses. Participants remained on ART during study treatment, and, therefore, impact on viral load kinetics was not expected. There were no virological failures (HIV RNA >200 copies/mL) during the study.

PD-1 blockade in oncology (25- to 250-fold higher dose than used in this study) has been associated with a proliferative burst as measured with Ki67 marker on CD8^+^ T cells and an expansion of CD8^+^CD45RA^−^CD62L^−^ effector memory T cells within 4 weeks of treatment initiation [18, 19]. The impact of immune checkpoint inhibitors on HIV reservoir latency reversal was examined in a substudy of the AIDS Malignancy Consortium 095 Study in which PWH-1 with advanced malignancies were assigned to nivolumab (anti–PD-1) or nivolumab plus ipilimumab (anti–CTLA-4) [21]. In that study, anti–PD-1 alone showed no effect on HIV-1 latency, but the combination induced a modest increase in cell-associated unspliced HIV-1 RNA. The Cancer Immunotherapy Trials Network 12 (CITN-12; NCT02595866) study evaluated the safety and anticancer activity of pembrolizumab (anti–PD-1) in PWH with cancer receiving ART [8]. In CITN-12, a 1.65-fold increase in plasma HIV RNA was observed with pembrolizumab treatment, suggesting a potential for very modest HIV latency reversal with anti–PD-1 monoclonal antibodies [8]. In the present study with low-dose budigalimab, there was no evidence of the typical proliferative burst that is associated with PD-1 blockade or evidence of latency reversal post budigalimab dosing in the background of viral control with ART. It is hypothesized that the lack of biomarker changes was owing to low antigenemia required to engage T-cell receptors and activate T cells, suggesting that budigalimab dosing with analytical treatment interruption may be required to elicit T-cell responses. A separate associated phase 1b study (NCT04223804) assessed viral load kinetics, safety, and PK/PD of a multidose regimen of budigalimab during a closely monitored analytical treatment interruption [17]. In this study, delayed viral rebound and/or evidence of off-ART viral control was observed in 6/9 participants completing treatment with four 10 mg biweekly doses, with 2 participants remaining off ART to study end (204 and 252 days).

In conclusion, this Phase 1b study of a single dose of budigalimab administered by SC or IV routes of administration provided findings that support an acceptable safety profile appropriate for the use of budigalimab in PWH at the studied doses, with favorable PK/pharmacodynamic profiles at 20 mg SC or 10 mg IV. Further evaluation of budigalimab as a potential component of treatment for HIV is warranted, and a phase 2 study is underway (NCT06032546).

## Supplementary Material

jiag142_Supplementary_Data

## Supplementary Data

Supplementary materials are available at The Journal of Infectious Diseases online (http://jid.oxfordjournals.org/). Supplementary materials consist of data provided by the author that are published to benefit the reader. The posted materials are not copyedited. The contents of all supplementary data are the sole responsibility of the authors. Questions or messages regarding errors should be addressed to the author.
