# Supplementary material for: Safety, Pharmacokinetics, and Pharmacodynamics of Single-Dose Programmed Cell Death Protein 1 Inhibitor, Budigalimab, in People With HIV-1 With Antiretroviral Therapy–Suppressed Viral Load
Source: J Infect Dis. 2026 Apr 7;234(2):e355–62. doi: 10.1093/infdis/jiag142 (PMC13537148; doi:10.1093/infdis/jiag142)

Supplementary Materials

Supplementary Methods

Cell-associated unspliced RNA

Quantitative droplet digital polymerase chain reaction (ddPCR) assay targeting conserved regions of unspliced HIV-1 RNA within the gag gene was used to measure HIV latency reversal at weeks 1 and 2 postdosing. Peripheral blood mononuclear cells (PBMCs) were isolated from anticoagulated blood by Labcorp. CD4+ T cells were isolated from PBMCs using a RosetteSep (StemCell Technologies, Vancouver, Canada) CD4+ T Negative Isolation Kit. RNA was extracted using a QIAamp® Viral RNA Mini Kit (Qiagen, Germantown, MD, USA), following the manufacturer's protocol, and eluted in water. RNA concentration was measured by spectrophotometry (Nanodrop, Qubist 4.0, Thermo Fischer Scientific, Waltham, MA, USA). RNA used as positive control for ddPCR was generated via in vitro transcription (IVT) using MEGAscript T7 Transcription Kit (Ambion, Thermo Fischer Scientific) kit from plasmid encoding the entire HIV gag (pNL4-3, AIDS Reference Research Reagent Repository) linearized with Not I using manufacturer's protocol.

Following Bio-Rad One-Step RT-ddPCR Advanced Kit (Hercules, CA, USA) manufacture recommended protocol, ddPCR reaction was set up using isolated RNA from PBMCs. Each 20-µl reaction in triplicate contained 1.5 µl, reverse transcriptase one-step RT-ddPCR Supermix, 300 mM DTT, 900 nM primers targeting HIV-1 gag region (FWD 5'-TCAGCCCAGAAGTAATACCCATGT -3'; REV 5'- CACTGTGTTTAGCATGGTGTTT -3'), 250 nM FAM-labeled gag probe (5'-ATTATCAGAAGGAGCCACCCCACAAGA-3'), and 5 µg of RNA template [22]. QX ONE ddPCR System is designed to deliver automatic, multiplexed

digital PCR. The QX ONE is an integrated system which seamlessly combines the standard ddPCR workflow of droplet generation, thermal cycling, and droplet reading into 1 instrument. The 20 µl reaction mixture was loaded into the Bio-Rad QX ONE device and downstream ddPCR processes were automatically performed within the QX ONE. Thermal cycling conditions were as follows: 50 °C for 60 minutes, 10 minutes at 95 °C, 40 cycles each consisting of a 30-second denaturation at 94°C followed by 40 cycles of denaturation at 95 °C for 30 seconds, annealing/extension at 56 °C for 1 minute, with ramping control of 2 °C/s, enzyme heat kill at 98 °C for 10 minutes, then held at 4 °C. At the end of polymerase chain reaction cycling, QX ONE generated raw fluorescence data.

Raw fluorescence data for each well were exported from the manufacturer's software (Bio-Rad QuantaSoft v.1.2) for analysis. The number of template copies per unit volume (μ) was estimated from the number of positive events (n) detected in the corresponding channel and the number of total accepted droplets (N) by maximum likelihood. The distribution of templates within a drop was assumed to follow a Poisson distribution, and the number of positive droplets was assumed to follow a binomial distribution. CIs were estimated under the same assumptions. The droplet size was assumed to be 0.91 nL, consistent with the instrument manufacturer's software. Data were plotted using GraphPad Prism version 10.1.2 for Windows (San Diego, CA, USA).

Flow cytometry

T-cell activation and proliferation was measured at Labcorp, using a validated assay that included 3 panels at weeks 1 and 2 postdosing. Panel 1 included CD45RO, Granzyme B, human leukocyte antigen (HLA)-DR, markers antigen Kiel 67 (Ki67), CD4, CCR7, CD8, and CD3; panel 2 included CD45RO, CD25, HLA-DR, Ki67, CD4, CD127, CD8, and CD3; and panel 3

included CD3, CD4, CD8, and isotype controls. Data were analyzed using GraphPad Prism version 10.1.2 for Windows (San Diego, CA, USA).

Elispot assay

Peripheral blood mononuclear cells (PBMCs) were isolated from anticoagulated blood by Labcorp. The ELISpot assay was performed to quantify IFNγ-secreting cells. ELISpot plates (human IFN-γ – ALP, Mabtech) were prepared per manufacturer's instructions. Briefly, plates were washed 3 times with sterile PBS (200µL/well) and subsequently conditioned with 200µL/well complete RPMI (c-RPMI: RPMI-1640, 10% FBS, Pen-Strep, 20 mM HEPES) for 30 minutes. PBMCs (500,000 per well) were mixed with PepTivator CMV pp65 Peptide (Miltenyi Biotec) or PepMix HIV-1 Gag Ultra Peptide (JPT) at a concentration of 0.1 µg/mL per reaction, or left unstimulated. Plates were incubated at 37°C with 5% CO2 for 18 hours. Subsequently, cells were removed and the plates were washed 5 times with PBS, 200µL/well. Detection antibody, 7-B6-ALP (Mabtech), was diluted per manufacturer's instructions, added to the plates and incubated for 2 hours at room temperature. The plates were washed 5 times with PBS, 200µL/well. Substrate, BCIP/NBT-plus (Mabtech), was prepared per manufacturer's instructions, added to the wells and the resulting spots were developed for 10-15 minutes. The plates were subsequently washed in tap water to stop the reaction, dried, and the spots were counted on Elispot reader (S6 entry M2, Immunospot by CTL).

References

22. Kiselinova M, Pasternak AO, De Spiegelaere W, Vogelaers D, Berkhout B, Vandekerckhove L. Comparison of droplet digital PCR and seminested real-time PCR for quantification of cell-associated HIV-1 RNA. PLoS One, **2014**; 9: e85999.

Supplementary Table

Supplementary Table 1. Dose-normalized C_{max} and AUC_t of budigalimab after single 10 mg and 20 mg SC doses: geometric mean (mean, %CV)

PK parameter	Budigalimab 10 mg SC (N=7)[a]	Budigalimab 20 mg SC (N=7)[a]
C_{max}/dose(µg/mL/mg)	0.04 (0.05, 74.0)	0.08 (0.08, 20.3)
AUC_t/dose (µg·day/mL/mg)	0.57 (0.895, 93.9)	1.70 (1.74, 23.3)

AUC_t, area under the serum concentration–time curve from time 0 to the time of the last measurable concentration; C_{max}, maximum observed serum concentration.
[a]One participant in the 10 mg SC and 20 mg SC groups had all budigalimab concentrations below the level of quantitation and was excluded from the analysis.

Supplementary Figures

Supplementary Figure 1. Dose-normalized C_{max} and AUC_t of budigalimab after single 10 mg and 20 mg SC doses: arithmetic mean (±SD). AUC_t, area under the serum concentration-time curve from time 0 to the time of the last measurable concentration; C_{max}, maximum observed serum concentration; IV, intravenous; SC, subcutaneous.

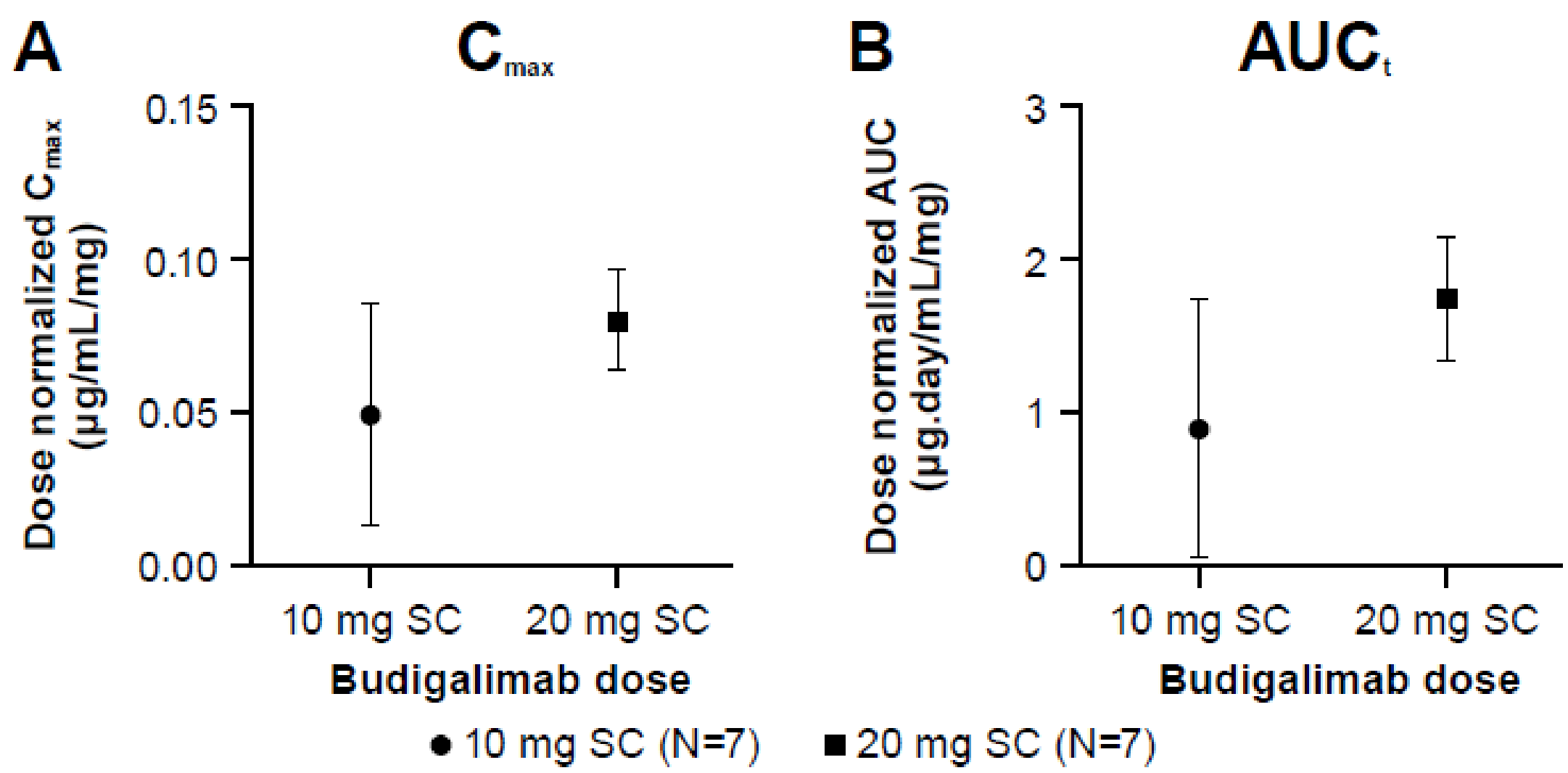

Supplementary Figure 2. Mean + SD budigalimab serum concentration–time profiles stratified by treatment-emergent ADA: (**A**) linear scale and (**B**) semi-log scale. *1 participant in the 10-mg SC group had all budigalimab concentrations below the level of quantitation and was excluded from the analysis. This participant was ADA-negative. ADA, antidrug antibody; IV, intravenous; SC, subcutaneous.

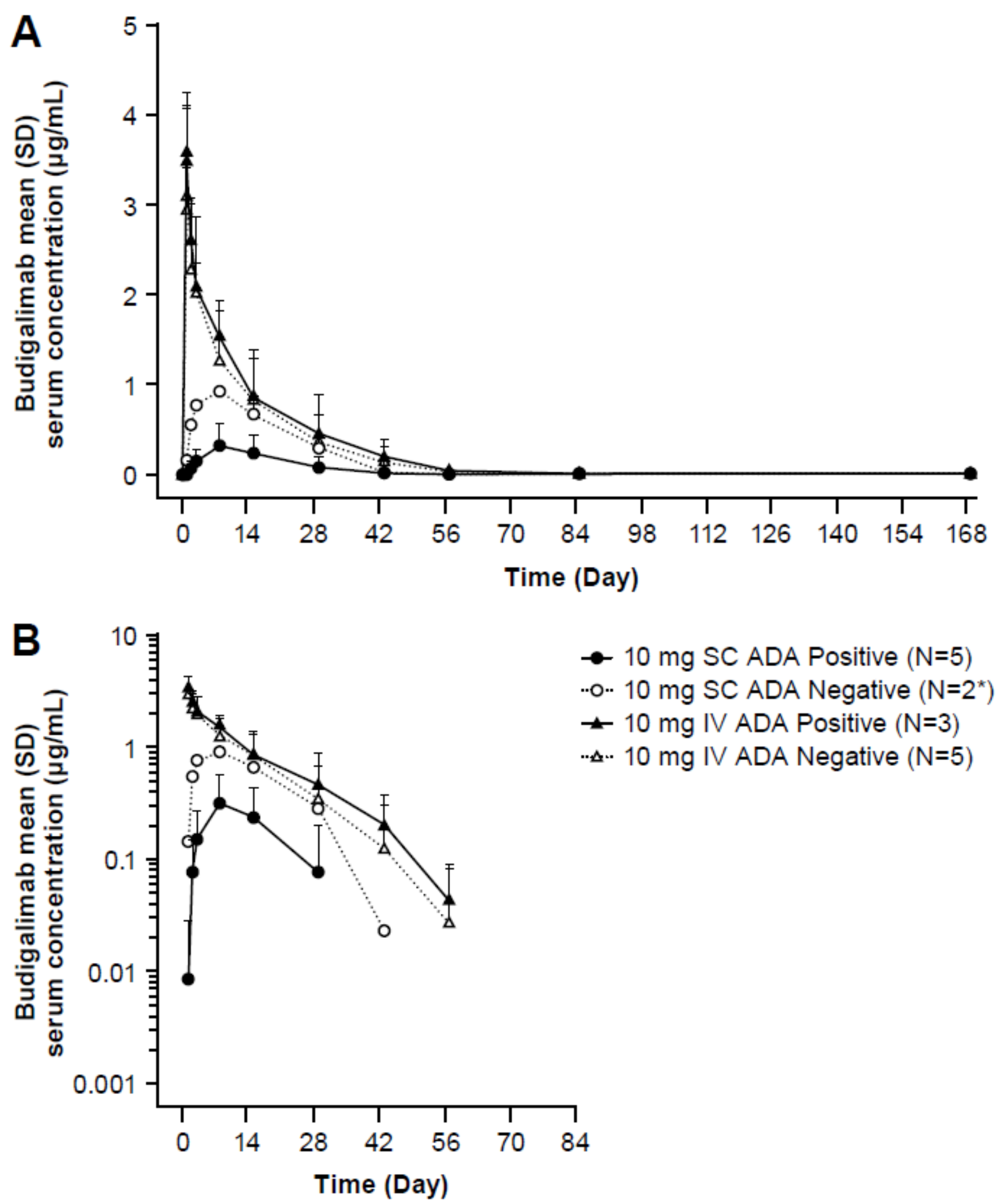

Supplementary Figure 3. T cell proliferation post budigalimab or placebo dosing. Frequency of CD8+ T memory cells (CD45RO+) expressing proliferation marker Ki67 at baseline, and W1 and W2 post dosing are shown. Connecting lines indicate means for each dose group. The frequency of Ki67 expressing cells for participants in: PBO arm (N=8) were 0.8±0.5 %, 0.7±0.4%, 0.8±0.3% at baseline, W1, and W2, respectively; 10mg SC arm (N=7) were 0.9±0.5 %, 0.8±0.3%, 0.8±0.5% at baseline, W1, and W2, respectively; 20mg SC arm (N=8) were 0.8±0.4 %, 0.9±0.4%, 1.2±0.7% at baseline, W1, and W2, respectively; 10mg IV arm (N=8) were 0.8±0.6 %, 1.2±0.6%, 1.1±1.0 % at baseline, W1, and W2, respectively. Frequency of Ki67 expressing cells not significantly different at W1 or W2 relative to baseline in each of the treatment arms by Mann-Whitney test (P value > 0.1). The population shown in this figure is representative. IV, intravenous; SC, subcutaneous, W1; week 1; W2, week 2.

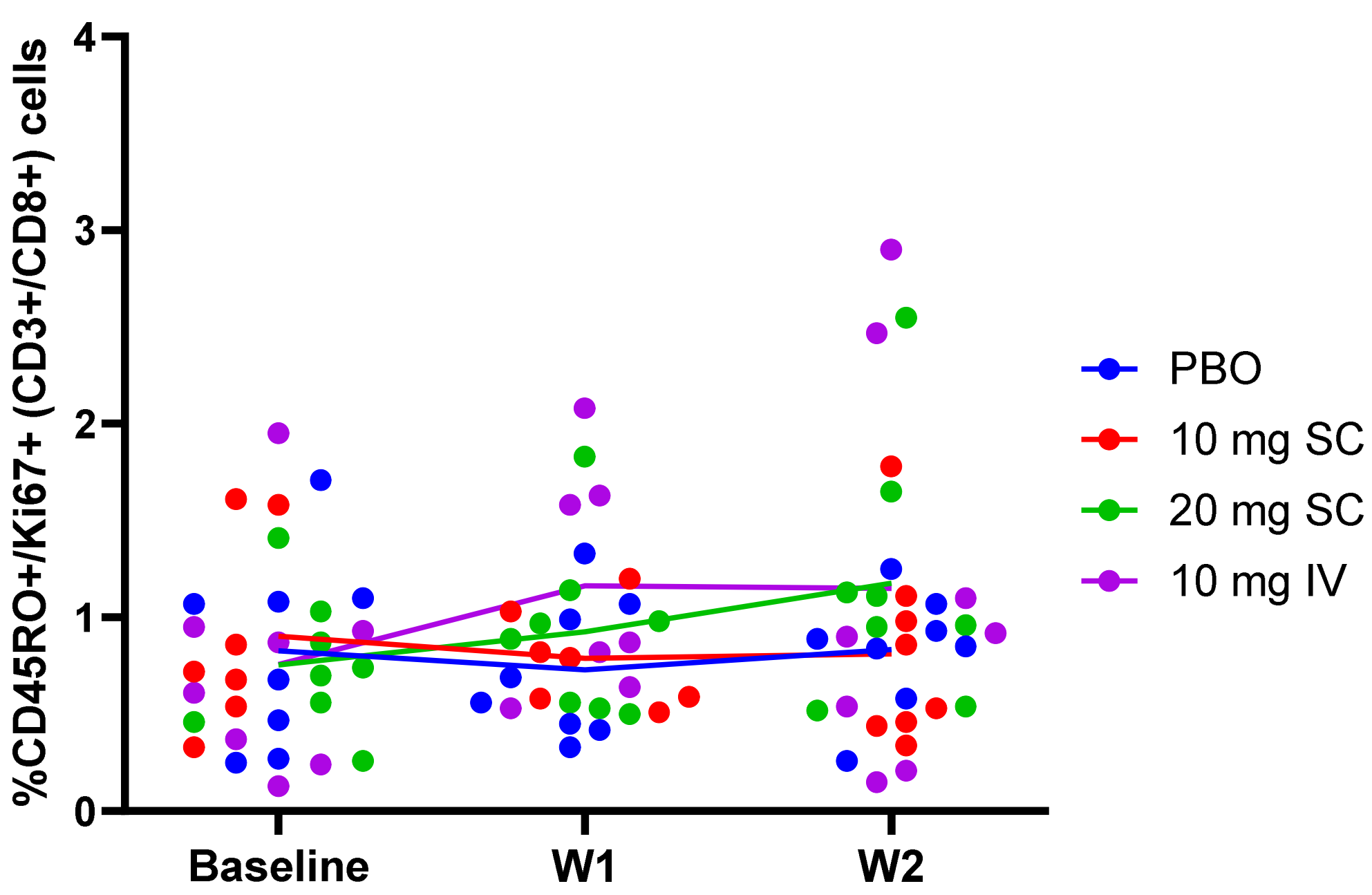

Supplementary Figure 4. Lack of impact of budigalimab dosing in the presence of ART on HIV latency. Cell-associated unspliced RNA per million CD4+ Tcells were measured at baseline (before first dose), W1, and W2 with primers targeting conserved region in gag. Connecting lines indicate means for each dose group. Cell-associated unspliced RNA per million CD4+ T cells for participants in: PBO arm (N=8) were 128±105, 117±96, 140±81 copies/ million CD4+ T cells at baseline, W1, and W2, respectively; 10mg SC arm (N=8) were 168±150, 185±198, 193±189 at baseline, W1, and W2, respectively; 20mg SC arm (N=8) were 126±71, 182±113, 179±93% at baseline, W1, and W2, respectively; 10mg IV arm (N=8) were 111±82, 116±9, 108±87 at baseline, W1, and W2, respectively. Differences in cell-associated unspliced RNA were not significantly different at W1 or W2 relative to baseline in each of the treatment arms by Mann-Whitney test (P value > 0.1). ART, antiretroviral therapy; IV, intravenous; RNA, ribonucleic acid; SC, subcutaneous; W1, week 1; W2, week 2.

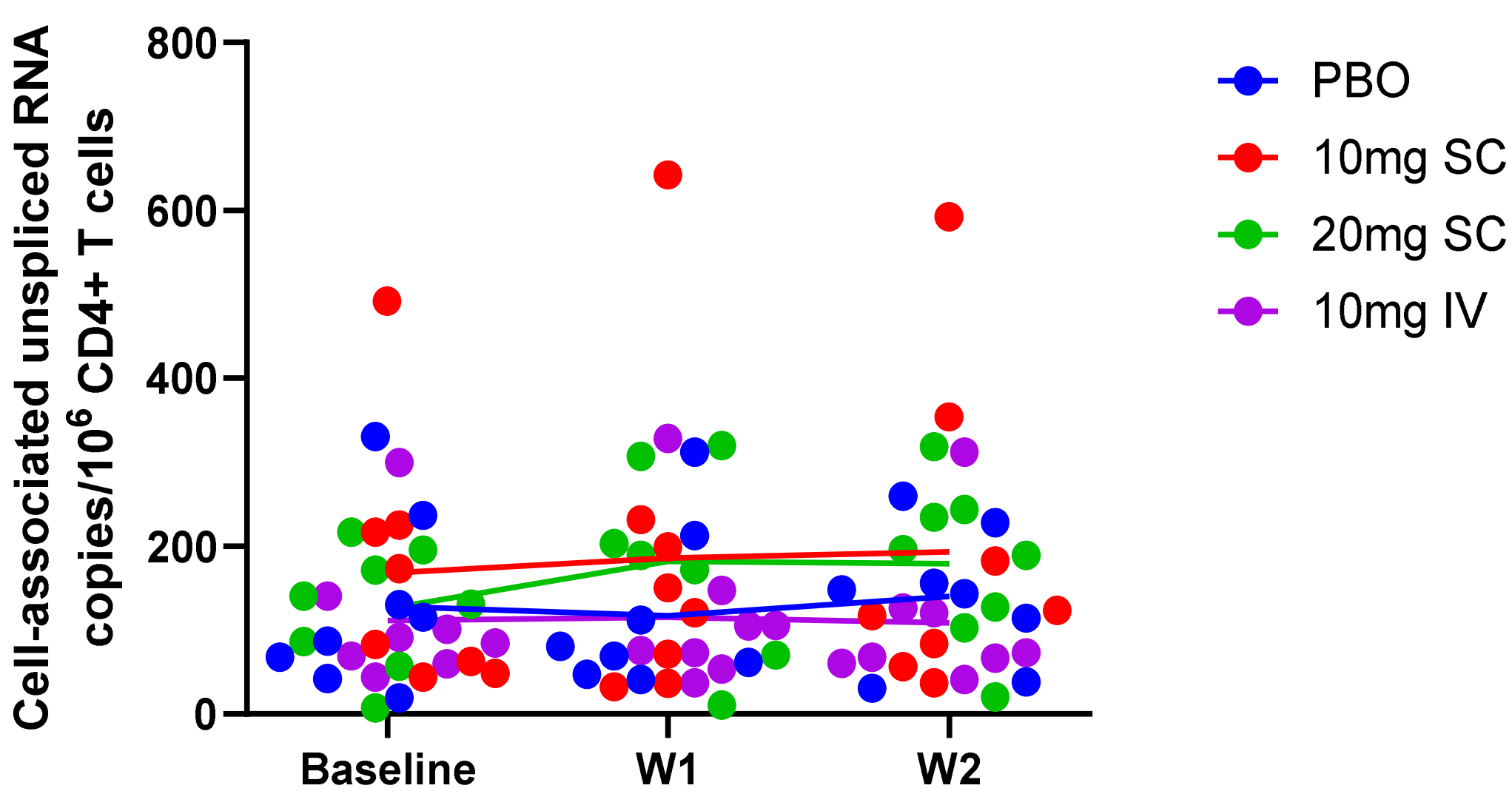

Supplementary Figure 5. Elispot assay measuring IFNγ response to stimulation of PBMCs with (a) gag peptide pool, and (b) CMV peptide pool. Bars indicate mean and SEM for each dose group. CMV, cytomegalovirus; IV, intravenous; PBMC, peripheral blood mononuclear cells; SC, subcutaneous, SEM; standard error of the mean; W1; week 1; W2, week 2.

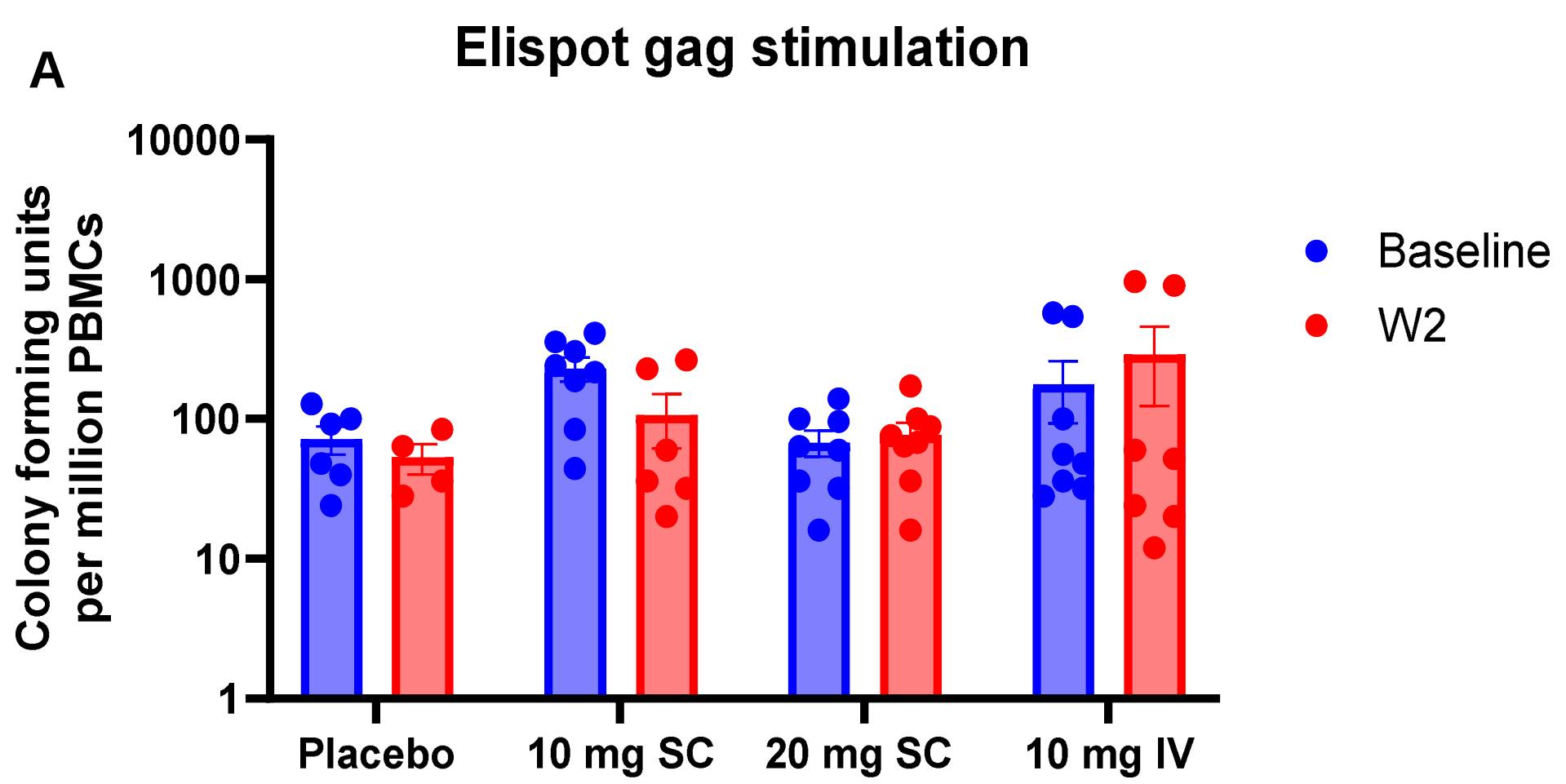

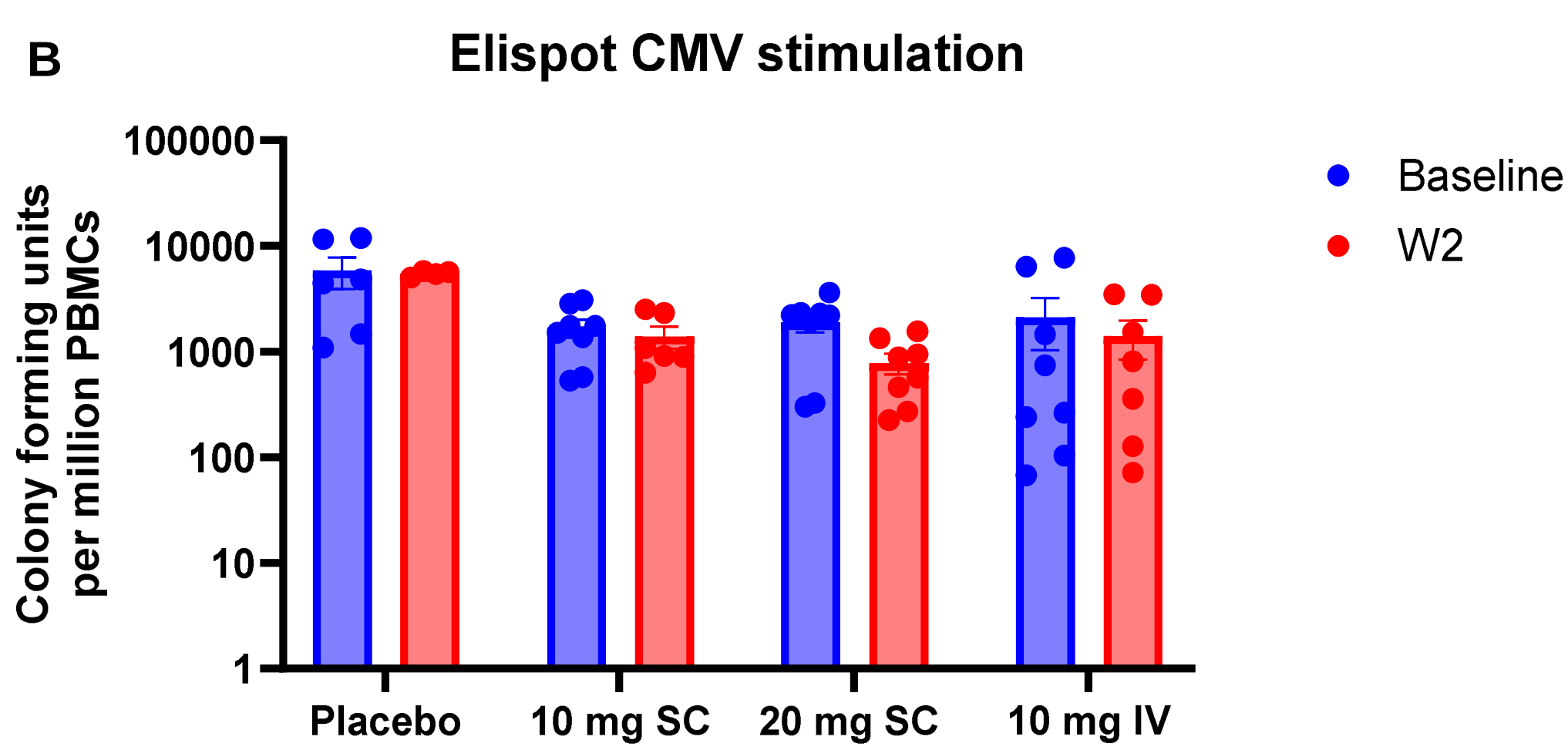

Supplement: Supplementary file 1 [file jiag142_supplementary_data.pdf]
